# Detection of Pb^2+^ in Tea Using Aptamer Labeled with AIEgen Nanospheres Based on MOFs Sensors

**DOI:** 10.3390/bios12090745

**Published:** 2022-09-09

**Authors:** Li Gao, Yixi Deng, Haolu Liu, King Solomon, Bianjiang Zhang, Huimei Cai

**Affiliations:** 1 School of Life Sciences, Jiangsu University, Zhenjiang 212013, China; 2 State Key Laboratory of Tea Plant Biology and Utilization, Anhui Agricultural University, No. 130 Changjiang West Road, Hefei 230036, China; 3 Nanjing Institute of Mechanization, Ministry of Agriculture, Nanjing 210014, China; 4 School of Food Science, Nanjing Xiaozhuang University, Nanjing 211171, China

**Keywords:** lead ions, fluorescent biosensors, metal-organic frames, tea

## Abstract

Tea is an important economic crop and health beverage in China. The presence of heavy metal ions in tea poses a significant threat to public health. Here, we prepared an aptamer biosensor labelled with AIEgen nanospheres to detect Pb^2+^ in tea. The dsDNA modified by amino and phosphoric acid was combined with the carboxylated AIEgen NPs to form AIEgen-DNA with a fluorescence group, which was then fixed to the surface of Zr-MOFs to quench the fluorescence of AIEgen NPs. At the same time, PEG was added to remove nonspecific adsorption. Then Pb^2+^ was added to cut the DNA sequences containing the cutting sites, and AIEgen NPs and part of the DNA sequences were separated from the Zr-MOFs surface to recover the fluorescence. By comparing the fluorescence changes before and after adding Pb^2+^, the detection limit of Pb^2+^ can reach 1.70 nM. The fluorescence sensor was applied to detect Pb^2+^ in tea, and the detection results showed that the tea purchased on the market did not contain the concentration of Pb^2+^ within the detection range. This study provides new insights into monitoring food and agriculture-related pollutants based on fluorescent biosensors.

## 1. Introduction

Tea is an important economic crop and healthy beverage in China, which has antioxidant, anti-tumour, hypoglycemic, and other effects [1]. With the increase of industrial and human activities, such as industrial and domestic sewage discharge, toxic heavy metal ions have become important pollutants, which can endanger human health throughout the food chain. Pb^2+^ is one of the toxic heavy metals. Even at very low concentrations, it can affect human health, causing severe damage to the brain, nervous system, hypertension, mental retardation, reproductive system, anemia, red blood cells, and kidneys [2]. It easily accumulates in plants (such as tea) and then cause harm to the human body through the food chain. Due to the significant threat of Pb^2+^ to public health, organisations worldwide have defined safety limits or maximum pollution levels for drinking water. For example, the maximum lead concentration in drinking water defined by the World Health Organisation (WHO) is 0.01 mg/L (48 nM) [3]. Therefore, it is of great significance to develop a highly sensitive and selective method for detecting lead ions in tea.

Up to the present, the methods used to detect heavy metal ions mainly include atomic absorption/emission spectrometry (AAS/AES) [4,5], mass spectrometry (MS) [6], inductively coupled plasma mass spectrometry (ICP-MS) [7,8], anodic stripping voltammetry (ASV) [9], high performance liquid chromatography (HPLC) [10], X-ray [11], etc. However, despite their high sensitivity and accuracy, these methods usually require specialized equipment, complex sample preparation, or time-consuming and laborious program operations that only professionals can perform. These limitations hinder the application of these technologies in clinical and field detection [12]. The emergence of biosensors provides a new detection method for life science research. Because of its characteristics of good selectivity, high sensitivity, fast analysis speed, low cost, and continuous online monitoring in complex systems, it has shown broad application prospects in the fields of biology, medicine, environmental monitoring, food, and military medicine, which has attracted the great attention of scientific researchers [13,14].

Among various sensor detection methods, fluorescent biosensors are favoured due to their high sensitivity, good selectivity, low sample consumption, and convenient operation [15]. Therefore, many fluorescent sensors based on functional materials, such as gold nanoparticles [16], silver nanoclusters [17], carbon nanomaterials [18], and silica nanoparticles [19], have been used for detection. Li et al. [20], combined sol-gel reaction, hydrothermal reaction, and carboxyl fluorescein (FAM) modified DNA bioaffinity adsorption to prepare a novel DNA-functionalized Fe_3_O_4_@TiO_2_NPs fluorescent “open” biosensor. The sensitivity and selectivity of the biosensor were evaluated by the fluorescence difference between DNA and Pb^2+^ before and after the reaction.

Aggregation-induced emission (AIE) is a special photophysical phenomenon opposite to aggregation-induced quenching. AIE fluorophore emits a high level of aggregation due to the restriction of intramolecular rotation. Aggregation-induced emission is an extraordinary photophysical phenomenon opposite to aggregation-induced quenching. Because of the restriction of intramolecular rotation, AIE fluorophore emits strongly in aggregation [21,22], and has been used in sensors [23], bioimaging [24], biotransfer [25], and therapeutics [26,27]. Researchers have recently found that coupling or adsorbing fluorescent molecules into nanoparticles can improve detection sensitivity [28]. According to morphological studies, Li et al. [29] designed an opportunity AIE emitter (AIEgen) of silica nanoparticles and discovered that the silica precursor polymerisation emits strong fluorescence. On the other hand, the internal spatial structure of polymer nanoparticles can limit the rotation of AIE molecules, so assembling AIEgen into nanoparticles can also emit fluorescence. Up to now, AIEgen has been widely used in virus detection [30], cell secretion localisation [31], and antibiotic detection [32].

Metal–organic frameworks (MOFs) are porous inorganic–organic hybrid materials with periodic network structures formed by self-assembling metal ions and organic ligands. Due to their structural diversity, flexibility and variability, and unique chemical and porous structures, MOFs have broad application prospects in gas storage, adsorption separation, catalysis, drug delivery, and biosensors [33,34]. Some metal ions, such as Zn^2+^ and Cu^2+^, are usually used as coordination centres and open sites. They have inherent fluorescence quenching characteristics and can be used as ideal quenching materials in fluorescent biosensors [35,36].

**Figure 1:**
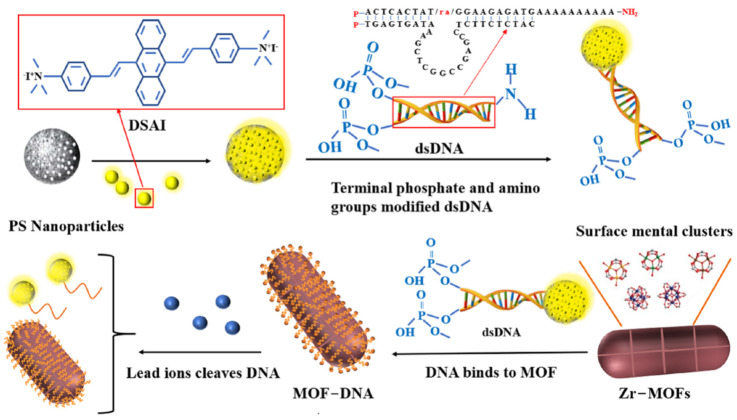
Schematic diagram of AIEgen nanosphere-labelled aptamer combined with Zr-MOFs for detection of Pb^2+^.

The binding of phosphate in the DNA skeleton or simulated body fluid to the metal of a MOF has been demonstrated to contribute to the decomposition of MOF connectivity. Wang et al. [37] used phosphate-modified oligonucleotides at the end to prove that MOF can bind DNA through metal–phosphate coordination and retain the integrity of the MOF structure, which has been used for the detection of Hg^2+^, Ag^+^ [36,37]. In this study, as shown in Figure 1, the phosphate group (-P) was modified at the end of double-stranded DNA (dsDNA) to fix it on the surface of Zr-MOFs. The other end of the DNA sequence containing the Pb^2+^ specific cleavage site modifies the amino group (-NH_2_) so that it can bind to the carboxylated AIEgen NPs synthesised by polystyrene nanospheres (PS NPs) and aggregation-induced luminescence materials (DSAI). A DsDNA (AIEgen-DNA) with a fluorescent group is formed. Subsequently, PEG was introduced into the sensing system to remove the interference matrix adsorption and further improve the sensitivity. Then, Zr-MOFs were combined with dsDNA to quench the fluorescence of AIEgen nanoparticles (AIEgen NPs). When Pb^2+^ was added, the DNA sequence containing the cleavage site was cut, and the conformation was changed. AIEgen NPs and some DNA sequences were separated from the surface of Zr-MOFs to emit light. By comparing the fluorescence changes before and after adding Pb^2+^, the detection of Pb^2+^ concentration was realised.

## 2. Materials and Methods

### 2.1. Chemical Reagents and Experimental Materials

The aptamer sequence was selected according to the relevant reports of Teh et al. [38].

Aptamer 1, 5′ -P-ACT CAC TAT/ra/GGA AGAGAT GAA AAA AAA AA-NH_2_-3′.

Aptamer 2, 5′-CATCTCTTCTCCGAGCCGGTCGAA ATA GTG AGT-P-3.′

The design mentioned above of the accounting adaptation sequence was synthesized by Bioengineering (Shanghai, China) Co., Ltd., purified by HPLC and verified by mass spectrometry. Sodium dodecyl sulfonate (SDS) was ordered to Sigma, polystyrene (P.S.) nanoparticles were ordered to Suzhou Derivative Biotechnology Co., Ltd. (Suzhou, China), dichloromethane was ordered to Aladdin Reagent (Shanghai, China) Co., Ltd., tetrastyrene monoquaternary ammonium salt (DSAI) was purchased from Xi’an Qiyue Biotechnology Co., Ltd. (Xi’an, China), polyethylene glycol (PEG) was purchased from Beijing Bailingwei Biotechnology Co., Ltd. (Beijing, China) A 0.22 μm water filter was purchased from Changde Bickerman Biotechnology Co., Ltd. (Changde, China), and Zr-MOFs/PCN-222 was purchased from Xi’an Qiyue Biotechnology Co., Ltd. (Xi’an, China).

### 2.2. Laboratory Apparatus

A Bio-Tek Synergy H4 multifunctional microplate reader was used to collect and record all fluorescence spectra under the excitation of 430 nm. A high-resolution transmission electron microscope (model: JEM-2100 (H.R.)) generates TEM images at 200 kV; FD-1A-50 freeze-drying agent freeze-dries the sample to obtain a powder solid, and nexus 670 FT-IR was scanned in projective mode by Fourier transform infrared (FT-IR) spectroscopy. Treatment of the sample solution in E.P. tube with H1650-W high-speed centrifuge; ultrapure water was prepared using UPH water purifier (18.25 Mcm); DF-101S constant temperature heating magnetic stirrer.

### 2.3. Aptamers Labelled with AIEgen Nanospheres (AIEgen-DNA)

Preparation of AIEgen nanospheres: AIEgen nanospheres were prepared according to Hu et al [39]. A total of 100 mg of polystyrene nanospheres were dissolved in 10 mL of ultrapure water (containing 0.25%) SDS to obtain a well-dispersed solution. 0.5 mL of DSAI (mass fraction of 10%) dissolved in CH_2_Cl_2_ solution was added. The solution was subjected to ultrasonication for 1 min and then stirred at 40 °C to rotate and evaporate CH_2_Cl_2_. The obtained AIEgen nanospheres were washed with ultrapure water for three times to remove SDS and then centrifuged with ethanol many times until no fluorescence was observed in the supernatant. Finally, the solid was obtained by freeze-drying. A certain amount of solid solution was dissolved in ultrapure water to prepare an ultrasonically uniform final concentration of 2 mg/mL solution.

To obtain hybrid dsDNA (5 μM), the same concentrations of DNA1 and DNA2 were mixed in equal volumes, thoroughly mixed, and placed at 4 °C for overnight reaction. Preparation of AIEgen-DNA tagged by AIEgen nanospheres. Firstly, the carboxylated AIEgen NPs were prepared by referring to the method of Sun et al [40]. A total of 0.6 g NaOH and 0.5 g chloroacetic acid were added to 5 mL AIEgen nanosphere solution with a diameter of 50 nm, respectively, and ultrasonicated in a water bath for 2 h. AIEgen NPs samples were activated with chloroacetic acid under strongly alkaline conditions to activate epoxy and ester groups and convert part of a hydroxyl group (-O.H.) into carboxylic acid (-COOH). After repeated centrifugation and purification, the solution was dried to obtain the carboxyl functionalised AIEgen nanosphere solid. Following that, carboxylated AIEgen nanospheres were prepared to a final concentration of 2 mg/mL and reacted with 30 L (50 nM) terminally modified amino (-NH_2_) dsDNA for 12 h at 4 °C. The unconjugated dsDNA was removed by centrifugation and dsDNA labelled with fluorescent nanospheres (AIEgen-DNA) were redispersed in the buffer.

### 2.4. Tea Treatment

To extract the lead ions in the extracted tea as much as possible and make the test results reliable, three different methods, including the high-temperature ash method, high-temperature oxidation method and ultrasonic immersion method, were used to process the purchased tea samples according to the relevant reports of Szymczycha et al [41,42].

Method I: Tea samples weighing 2.0 g were accurately weighed, dried and ground, and sieved through 70 mesh (212 μm). A total of 0.5 g in a porcelain crucible was put into a muffle furnace, and ashed at 500 °C. After 5 mL of high purity nitric acid was added to the ashes, a clear and transparent solution was heat and transferred to a 10 mL volumetric flask with ultrapure water at constant volume as a tea sample storage solution.

Method II: In a PTFE container, 0.5 g of the filtered tea sample was placed, and 6.0 mL of nitric acid and 1.0 mL of hydrogen peroxide were added to make a concentrated oxidation reagent. Then, it was put into autoclaves and kept at 300 °C for 8 h. After that, it was cooled to room temperature to obtain a light-yellow transparent liquid, which was quantitatively transferred to a 10 mL volumetric flask and diluted with ultrapure water.

Method III: 0.5 g of tea samples after screening was loaded into a 30 mL P.P. centrifuge tube, and 5 mL of ultrapure water was added. The resulting mixture was soaked in room temperature ultrasound for 2 h and then placed overnight. After centrifugation, the brown–green sample solution was separated from the insoluble residue and quantitatively transferred to a 10 mL volumetric flask. The ultrapure water was used as the reserve solution for tea samples. Before detection, all the above tea reserves were filtered with a 0.22 μm aqueous microporous membrane.

## 3. Result and Discussion

### 3.1. Characterisation of P.S. Nanospheres and AIEgen NPs

**Figure 2:**
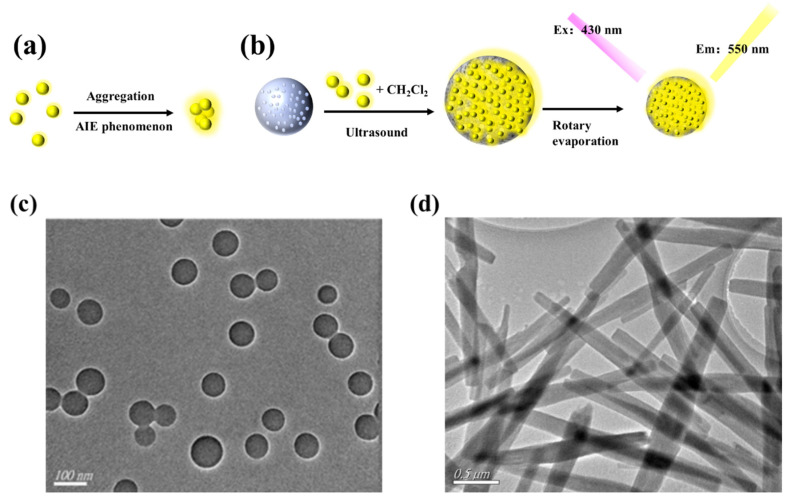

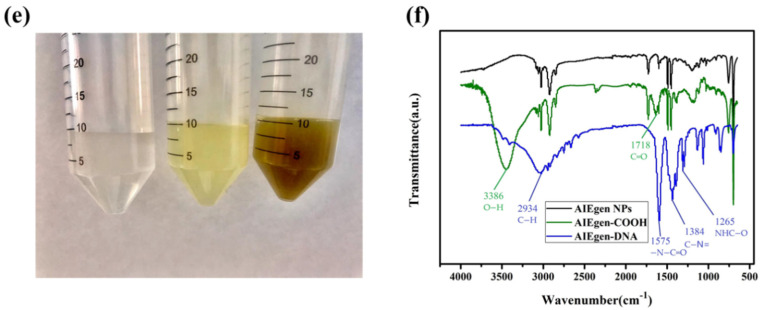
(**a**) The AIEgen principle (Left: no fluorescence without DSAI aggregation; high fluorescence with DSAI aggregation). (**b**) Synthesis of AIEgen nanospheres; (**c**) TEM image of AIEgen NPs; (**d**) TEM image of MOF; (**e**) Results of tea treated by three methods; (**f**) FT-IR spectra of AIEgen NPs modified with carboxyl functionalisation and DNA.

AIEgens emit faint luminescence when dispersed in solution but show strong fluorescence in the aggregated state, as shown in Figure 2a. Polystyrene nanospheres were swelled by sonication in CH_2_Cl_2_ for DSAI loading. Then CH_2_Cl_2_ was removed by rotary evaporation, and the pore size of the polystyrene nanosphere was restored to its original diameter. At this time, DSAI was sealed in the polystyrene nanosphere. The excitation and emission wavelengths of the prepared AIEgen NPs were 430 nm and 550 nm, respectively, as shown in Figure 2b. The morphology of AIEgen NPs was observed by transmission electron microscopy (TEM). The morphology of AIEgen NPs was observed by transmission electron microscopy (TEM). The morphology of AIEgen NPs was mostly spherical and well dispersed, with an average diameter of about 60 nm, as shown in Figure 2c, similar to Hu et al [39]. Figure 2d shows the morphology of ZR-MOFs used in this paper under TEM, which are primarily rod-shaped and uniformly dispersed in the solution. Figure 2e shows that, from left to right, tea solutions obtained by three methods, namely, high-temperature ashing, high-temperature oxidation, and ultrasonic soaking, are presented as transparent colours, light-yellow colours, brown, and green colours. Figure 2f shows the FT-IR spectra of AIEgen NPs, AIEgen -COOH, and AIEgen-DNA. In the FT-IR spectrum, it is observed that the stretching vibration absorption of several free carboxylic acids O-H of AIEgen -COOH is located at ~3550 cm^−1^. Due to the formation of dimers, the carboxylic peak is shifted to the direction of low wave number, the wave peak is skewed to ~3386 cm^−1^, and the broad and scattered peak is formed at ~3700–3000 cm^−1^. A free carboxylic acid’s C=O stretching vibration is located at ~1718 cm^−1^, indicating that AIEgen NPs are carboxylated successfully. AIEgen -DNA has characteristic peaks at ~2934 cm^−1^, ~1575 cm^−1^, ~1265 cm^−1^, and ~1384 cm^−1^, which are the characteristics of C-H, -N-C=O, C-N=, and NHC=O stretching, respectively. The results indicate that AIEgen -DNA was successfully synthesised.

### 3.2. Experimental Condition Optimisation

In order to achieve the best conditions for Pb^2+^ detection, the experimental conditions were optimised, including AIEgen-DNA concentration, Zr-MOFs concentration, quenching agent Pb^2+^ reaction time, and PEG concentration.

#### 3.2.1. Optimal Concentration Selection of AIEgen-DNA

**Figure 3:**
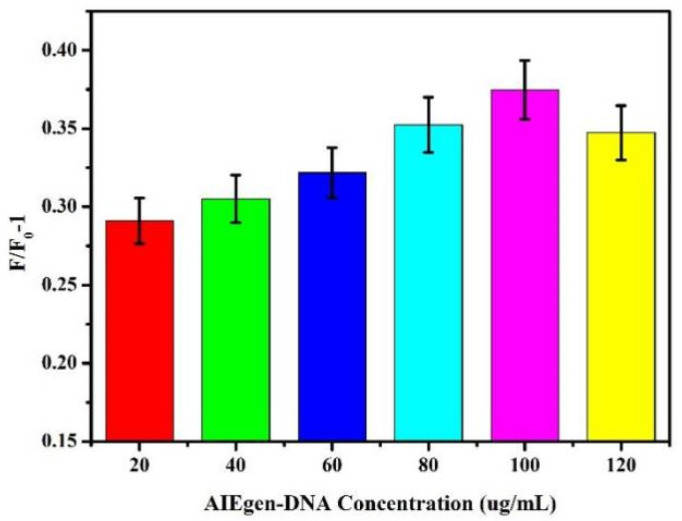
Effect of different concentrations of AIEgen-DNA on fluorescence intensity changes.

Different concentrations of AIEgen-DNA were selected. The final concentration of 50 nM dsDNA was added to the brown centrifuge tube containing 500 μL of ultrapure water. AIEgen NPs with different final concentrations (20 μg/mL, 40 μg/mL, 60 μg/mL, 80 μg/mL, 100 μg/mL, and 120 μg/mL) were added to the centrifuge tube solution. The mixture was evenly mixed and allowed to react overnight at 4 °C. Subsequently, under 13,000 rpm for 30 min, the supernatant was removed after centrifugation, and then ultrapure water was added to 500 μL. The final concentration of Zr-MOFs was 30 μg/mL. After mixing evenly, the mixture was kept at room temperature for 25 min, and 200 μL was transferred to the black 96-well plate. The fluorescence intensity at this time was measured to be F_0_. Subsequently, Pb^2+^ with the final concentration of 10 nM was added to 300 μL of the remaining liquid. After the reaction at room temperature for 40 min, 200 μL was absorbed into the black 96-well plate, and the fluorescence intensity at this time was measured to be F. The signal output value was calculated according to the fluorescence intensity variation formula F/F_0_-1, where F_0_ and F represent the fluorescence intensity of the sensor system based on AIEgen-DNA at 550 nM before and after the addition of Pb^2+^, respectively. As shown in Figure 3, F/F_0_-1 gradually increased as AIEgen-DNA concentration increased. When the concentration of AIEgen-DNA reached 100 μg/mL, F/F_0-_1 reached its maximum.

#### 3.2.2. Optimal Concentration of dsDNA

**Figure 4:**
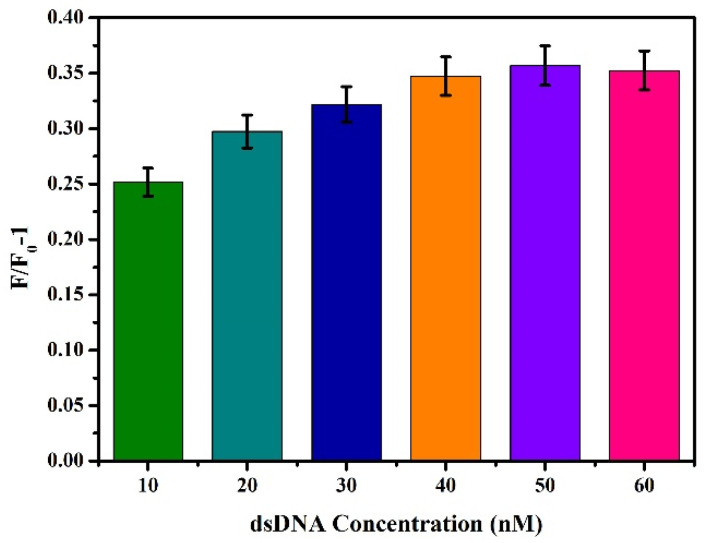
Effects of different concentrations of dsDNA on fluorescence intensity.

On the basis of the above conditions, the concentration of dsDNA was optimised, and different final concentrations (10 nM, 20 nM, 30 nM, 40 nM, 50 nM, and 60 nM) of dsDNA were added to the reaction system, and the change value of fluorescence intensity F/F_0_-1 was determined by referring to Section 3.2.1 for the other steps. As shown in Figure 4, F/F_0_-1 gradually increased with the increase of dsDNA concentration. When dsDNA concentration increased to 50 nM, F/F_0_-1 reached the maximum. At this time, if dsDNA concentration was increased again, F/F_0_-1 will not increase. The results indicate that the carboxylated AIEgen NPs have bound DNA to the maximum extent, and further increasing the dsDNA concentration will cause material waste and increase the background signal. Therefore, 50 nM was chosen as the optimal dsDNA concentration.

#### 3.2.3. Optimal Concentration of Zr-MOFs

**Figure 5:**
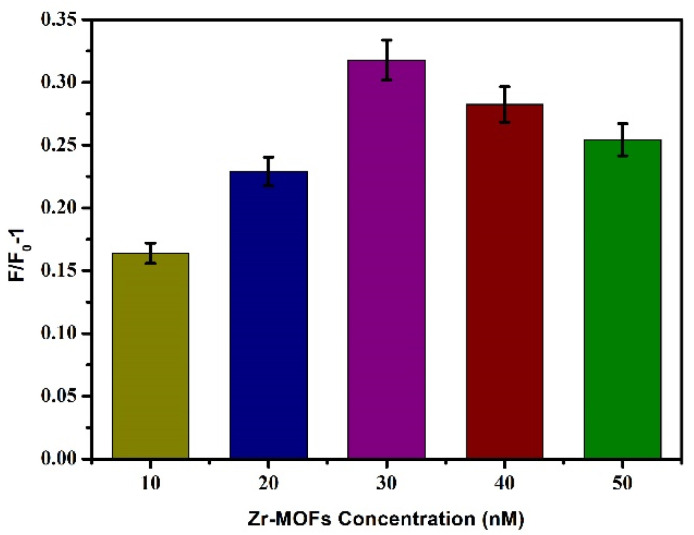
Effects of different concentrations of Zr-MOFs on fluorescence intensity.

Based on the above conditions, the concentration of the quencher was optimised, and different final concentrations (10 μg/mL, 20 μg/mL, 30 μg/mL, 40 μg/mL, and 50 μg/mL) of Zr-MOFs were added to the reaction system. The other steps referred to Section 3.2.1, and the fluorescence intensity change value F/F_0_-1 was determined. As shown in Figure 5, with the increase of Zr-MOFs concentration, F/F_0_-1 also gradually increased, and when the concentration reached 30 μg/mL, F/F_0_-1 reached its maximum. When the concentration exceeded 30 μg/mL, F/F_0_-1 decreased with the increase of Zr-MOFs concentration. A low concentration of Zr-MOFs is not conducive to the covalent binding between dsDNA and Zr-MOFs, and a higher concentration of Zr-MOFs will affect the recovery of fluorescence intensity after adding Pb^2+^. Therefore, 30 μg/mL was selected as the optimal Zr-MOFs concentration in this experiment.

#### 3.2.4. Reaction Time Optimisation

**Figure 6:**
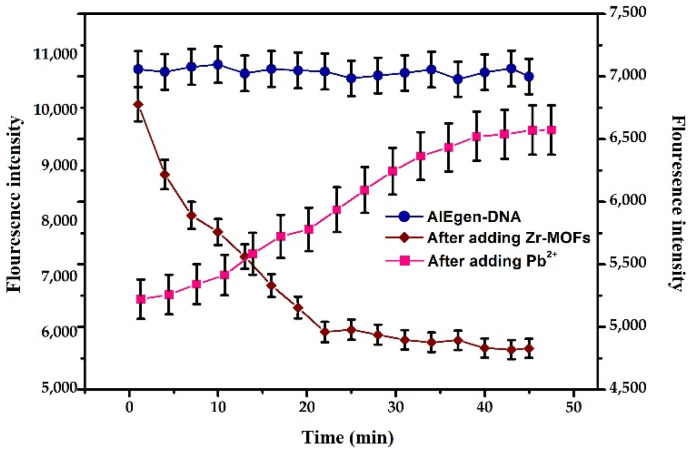
Changes in fluorescence intensity with time after adding different substances, the blue lines (AIEgen-DNA) and brown lines (After adding Zr-MOFs) correspond to the vertical axis on the left, and the pink lines (After adding Pb^2+^) correspond to the vertical axis on the right.

Based on the above optimal reaction conditions, the reaction time of adding the quencher and the tested substance was optimised. The AIEgen-DNA with the final concentration of 100 μg/mL, Zr-MOFs with a final concentration of 30 μg/mL and the added AIEgen-DNA, Zr-MOFs and Pb^2+^ were added to the reaction system, and the other steps were referred to in Section 3.2.1 for the kinetic detection of the reaction system. The changes in fluorescence intensity within 45 min after adding AIEgen-DNA, Zr-MOFs and Pb^2+^ were shown in Figure 6. The fluorescence intensity remained relatively stable within 45 min after adding AIEgen-DNA, showing a stable state. After adding Zr-MOFs, the fluorescence of AIEgen-DNA was quenched, and the fluorescence intensity decreased rapidly. After 25 min, the fluorescence intensity tended to be stable. Therefore, 25 min was selected as the quenching time in the subsequent experiments. After 10 nM Pb^2+^ was added, the quenched fluorescence began to recover, and the fluorescence intensity gradually increased. After 40 min, the fluorescence gradually stabilised. Therefore, 40 min was selected as the fluorescence recovery time after Pb^2+^ was added.

### 3.3. Sensitivity Detection

**Figure 7:**
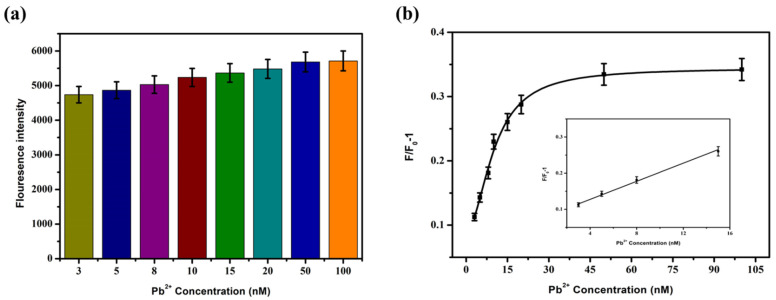
The fluorescence intensity changes (**a**) and F/F_0_-1 (**b**) after adding different concentrations of Pb^2+^ into the sensor.

Lead ions with different final concentrations (3 nM, 5 nM, 8 nM, 10 nM, 15 nM, 20 nM, 50 nM, and 100 nM) were added to the sensing system, and then the mixture was placed at room temperature for 30 min, concerning step Section 3.2.1. The fluorescence intensity at 550 nm was detected at an excitation of 430 nm. The sensitivity was detected according to the formula F/F_0_-1 for the change of fluorescence intensity. The results are shown in Figure 7. The fluorescence recovery degree was positively correlated with the Pb^2+^ concentration. The fluorescence intensity gradually increased with the increase in Pb^2+^ concentration. When the Pb^2+^ concentration was between 3 and 15 nM, the two showed a significant linear relationship, and the linear regression equation was F/F_0_-1 = 0.0125 × C_[pb^2+^]_ + 0.0774, R^2^ = 0.992. Based on 3N/S, the detection limit LOD was 2.75 nM.

### 3.4. Optimum Concentration of PEG

**Figure 8:**
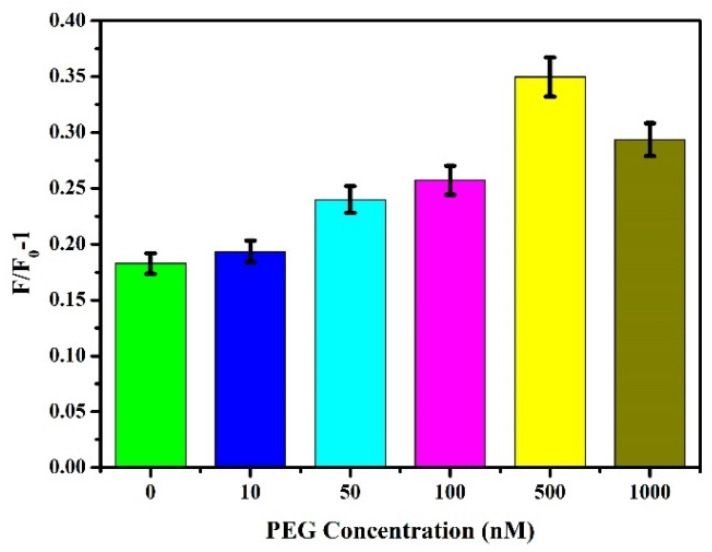
Effect of PEG with different concentrations on fluorescence intensity.

The nonspecific interaction between dsDNA and molecules other than AIEgen NP and Zr-MOFs led to the appearance of false-positive signals and a decrease in sensitivity. Studies have shown that surface modification of hydrophilic polymers can enhance the biocompatibility of synthetic polymers [43,44]. Among many polymers, PEG is a kind of affinity polymer with good protective performance and biocompatibility and has been widely used [45,46,47]. We used the lipophilic activity of PEG to interact with AIEgen NPs and Zr-MOFs to interfere with the nonspecific adsorption of dsDNA on Zr-MOFs and AIE surfaces, thereby improving the sensitivity of detection. Different concentrations (0, 10, 50, 100, 500, and 1000 nM) of PEG were added to the sensing system, and other steps were referred to in Section 3.2.1 to explore the effect of PEG concentration on the reaction system. As shown in Figure 8, as PEG concentration increased, F/F_0_-1 gradually increased, reaching its peak at 500 nM before weakening. A low concentration of PEG cannot achieve good antifouling performance. In contrast, a high concentration of PEG will affect the combination of AIEgen NPs and Zr-MOFs with dsDNA and the sensor’s sensitivity. Therefore, 500 nM was selected as the optimal PEG concentration.

### 3.5. Sensitivity Analysis after PEG Was Added

**Figure 9:**
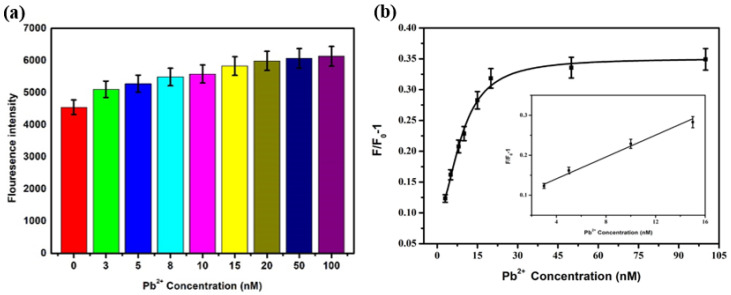
Under the condition of 500 nM PEG, (**a**) the fluorescence intensity and (**b**) the change of F/F_0-_1 after adding Pb^2+^ with different concentrations.

Under the condition of 500 nM PEG, Pb^2+^ with different concentrations (3 nM, 5 nM, 8 nM, 10 nM, 15 nM, 20 nM, 50 nM, and 100 nM) was added into the sensing system, and the steps were referred to as Section 3.2.4. The results were shown in Figure 9. The degree of fluorescence recovery depends on Pb^2+^ concentration, and the fluorescence intensity increases with the increase of Pb^2+^ concentration. When the concentration of Pb^2+^ was 3–15 nM, the F/F_0_-1 showed a significant linear relationship with it, and the linear equation was F/F_0-_1 = 0.0136 × C_[pb^2+^]_ + 0.0865, R^2^ = 0.981. Based on 3 N/S, the detection limit was 1.70 nM.

### 3.6. Selective Analysis

**Figure 10:**
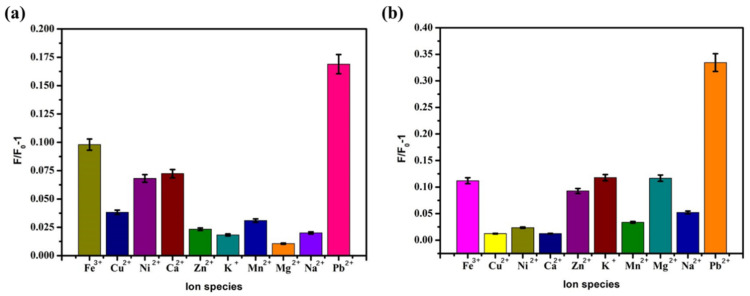
Optimal conditions, sensor selective detection results of (**a**) 5 nM Pb^2+^ and (**b**) 20 nM Pb^2+^.

Selectivity is an important indicator for evaluating the performance of Pb^2+^ sensors. In order to prove that the sensor has specificity for Pb^2+^, several different ions (Fe^3+^, Cu^2+^, Ni^+^, Ca^2+^, Zn^2+^, K^+^, Mn^2+^, Mg^2+^, Na^+^, and Pb^2+^) were selected for an interference test to evaluate the selectivity of the sensor. Under the optimal conditions, Pb^2+^ (5 nM and 20 nM) and other ions with the same concentration were added to the sensing system. Other ions with the same concentration were added to the sensing system. The experimental steps referred to Section 3.2.1 to measure the fluorescence intensity and compare F/F_0_-1. The detection results were shown in A and B of Figure 10. These can show the specificity of the sensing system for lead ions. When other ions were added to the reaction system, the fluorescence intensity did not increase. The results showed that the prepared sensor had good selectivity for Pb^2+^.

### 3.7. Stability of Practical Application

To prove the stability of the sensor in the actual detection, tap water in the reaction system replaced the ultrapure water in the reaction system. In the linear range, three concentrations of Pb^2+^ (3 nM, 5 nM, and 10 nM) were selected to carry out the standard recovery test. The specific operation steps were referred to as Section 3.2.1, and each group was repeated three times. The results are presented in Table 1. The recoveries of three samples were 100.10%, 104.11%, and 103.72%, respectively. The relative standard deviations are 0.729~2.649%, consistent with the relevant practical application requirements. It shows that the proposed method has good stability and practical application. It provides a theoretical basis for the precise detection of Pb^2+^ and has paramount practical significance.

### 3.8. Detection of Lead Ion in Tea

**Figure 11:**
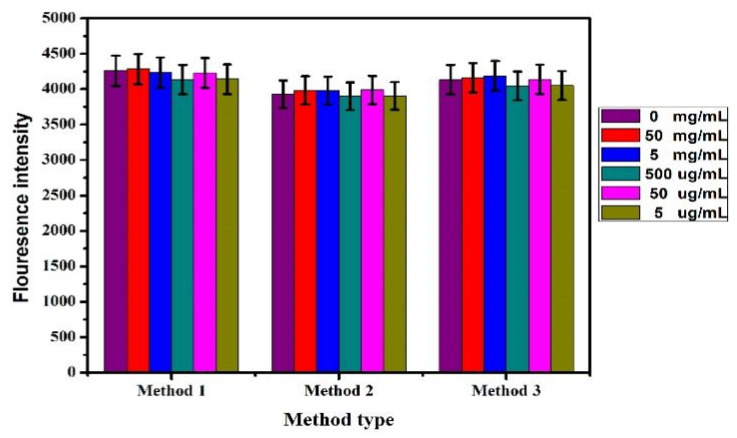
Detection of tea samples treated by different methods.

The Pb^2+^ content in tea was detected by the fluorescence biosensor prepared above. Three kinds of tea samples treated in 2.4 were added into the reaction system with different concentrations (50 mg/mL, 5 mg/mL, 500 μg/mL, 50 μg/mL, and 5 μg/mL). The detection results were shown in Figure 11. When different concentrations of tea treatment sample solutions were added to the sensing system, no obvious change in fluorescence intensity can be caused. This showed no lead ion was in the tea purchased on the market.

## 4. Conclusions

In summary, a fluorescent biosensor based on aptamer labelled with AIEgen NPs was prepared to detect Pb^2+^ in tea. A phosphoric acid group (-P) was modified at the end of double-stranded DNA (dsDNA) to fix it on the surface of Zr-MOFs. The amino group (-NH_2_) was modified at the other end of the DNA sequence containing the Pb^2+^-specific cleavage site, which was then combined with AIEgen NPs synthesised by polystyrene nanospheres (PS NPs) and aggregation-induced luminescence material (DSAI) and modified by carboxylation to form a fluorescent group dsDNA (AIEgen-DNA). Subsequently, Zr-MOFs were combined with dsDNA to quench the fluorescence of AIEgen NPs. When Pb^2+^ was added, the DNA sequence containing the cleavage site was cut, and the dsDNA conformation changed. AIEgen NPs and some DNA sequences were separated from the Zr-MOFs surface and glowed. By comparing the fluorescence changes before and after the addition of Pb^2+^, when the concentration of Pb^2+^ was 3–15 nM, F/F_0_-1 showed a significant linear relationship with it, and the detection limit was 2.75 nM. In order to further improve the sensitivity of the sensor, PEG was added to the sensor. When the concentration of Pb^2+^ was 3–15 nM, there was also a significant linear relationship, and the detection limit was 1.70 nM. The results showed that the sensor’s sensitivity was further improved after PEG was introduced into the sensor system, and then the sensor was applied to Pb in tea. The results showed that the sensor’s sensitivity was further improved after introducing PEG into the sensor system, and then the sensor was applied to detecting Pb^2+^ in tea. The detection results showed that the tea purchased on the market did not contain Pb^2+^ within the detection range.

## Figures and Tables

**Table 1:** Test results of standard recovery test (n = 3).

Samples	Added (ng/mL)	Obtained (ng/mL)	Recovery (%)	S.D. (%)	RSD (%)
1	3	3.003213	100.107108	0.0797	0.729
2	5	5.205697	104.11394	0.3771	2.649
3	10	10.372185	103.72185	0.4003	1.738

## Data Availability

Data available on request due to restrictions e.g., privacy or ethical. The data presented in this study are available on request from the corresponding author.
